# Skeletal Adaptation to Intramedullary Pressure-Induced Interstitial Fluid Flow Is Enhanced in Mice Subjected to Targeted Osteocyte Ablation

**DOI:** 10.1371/journal.pone.0033336

**Published:** 2012-03-07

**Authors:** Ronald Y. Kwon, Diana R. Meays, Alexander S. Meilan, Jeremiah Jones, Rosa Miramontes, Natalie Kardos, Jiunn-Chern Yeh, John A. Frangos

**Affiliations:** La Jolla Bioengineering Institute, La Jolla, California, United States of America; University of Notre Dame, United States of America

## Abstract

Interstitial fluid flow (IFF) is a potent regulatory signal in bone. During mechanical loading, IFF is generated through two distinct mechanisms that result in spatially distinct flow profiles: poroelastic interactions within the lacunar-canalicular system, and intramedullary pressurization. While the former generates IFF primarily within the lacunar-canalicular network, the latter generates significant flow at the endosteal surface as well as within the tissue. This gives rise to the intriguing possibility that loading-induced IFF may differentially activate osteocytes or surface-residing cells depending on the generating mechanism, and that sensation of IFF generated via intramedullary pressurization may be mediated by a non-osteocytic bone cell population. To begin to explore this possibility, we used the Dmp1-HBEGF inducible osteocyte ablation mouse model and a microfluidic system for modulating intramedullary pressure (ImP) to assess whether structural adaptation to ImP-driven IFF is altered by partial osteocyte depletion. Canalicular convective velocities during pressurization were estimated through the use of fluorescence recovery after photobleaching and computational modeling. Following osteocyte ablation, transgenic mice exhibited severe losses in bone structure and altered responses to hindlimb suspension in a compartment-specific manner. In pressure-loaded limbs, transgenic mice displayed similar or significantly enhanced structural adaptation to Imp-driven IFF, particularly in the trabecular compartment, despite up to ∼50% of trabecular lacunae being uninhabited following ablation. Interestingly, regression analysis revealed relative gains in bone structure in pressure-loaded limbs were correlated with reductions in bone structure in unpressurized control limbs, suggesting that adaptation to ImP-driven IFF was potentiated by increases in osteoclastic activity and/or reductions in osteoblastic activity incurred independently of pressure loading. Collectively, these studies indicate that structural adaptation to ImP-driven IFF can proceed unimpeded following a significant depletion in osteocytes, consistent with the potential existence of a non-osteocytic bone cell population that senses ImP-driven IFF independently and potentially parallel to osteocytic sensation of poroelasticity-derived IFF.

## Introduction

Bone has long been recognized to be a remarkably mechanosensitive organ, capable of undergoing rapid and profound structural alterations in response to relatively minute mechanical deformations. Since mechanical loading is one of the principal natural factors that determine bone strength [1], the bone mechanotransduction pathway is widely recognized as a strong potential target for the development of novel bone therapeutics [2]. A central goal in better understanding the mechanotransduction process is elucidating the physical signals driving skeletal adaptation to mechanical loading, and the cells responsible for sensing these signals.

Mechanical loading generates a number of physical signals within bone that have the potential to initiate structural adaptation (e.g., strain, electromagnetic fields, pressure, interstitial fluid flow, etc. [3]). Interstitial fluid flow (IFF) in particular has become widely recognized as a potent regulatory signal in bone [4]. For example, a variety of cells including osteocytes, osteoblasts, osteoclasts, osteoblastic/osteoclastic progenitors, and marrow stromal cells have been found to exhibit responses that support bone formation and/or inhibit resorption upon exposure to fluid flow *in vitro* [5], [6], [7], [8], [9], [10], [11]. *In vivo*, the capacity for IFF to regulate bone metabolism has been demonstrated in studies in which flow was largely decoupled from matrix strain [2], [12], [13], [14]. These studies suggest that adaptation to dynamic IFF can occur in cortical and trabecular bone in the presence of minimal tissue deformation at the site of adaptation.

Mechanical loading has been demonstrated to induce skeletal IFF through two distinct mechanisms that give rise to distinct spatial patterns of flow: poroelastic interactions within the lacunar-canalicular system (LCS) between the interstitial fluid and bone tissue [15], [16], and intramedullary pressurization [2], [17], [18], [19]. Whereas poroelastic interactions within the LCS are expected to give rise to IFF primarily within the lacunar-canalicular network, pressurization of the intramedullary cavity is expected to result in significant levels of IFF at the endosteal surface as well as within the LCS due to the gross outward pressure gradient from the intramedullary cavity to the periosteal surface [2], [12]. In this case, IFF derived from poroelastic interactions within the LCS would be expected to primarily expose osteocytes residing within the lacunar-canalicular network to flow, whereas intramedullary pressurization would be expected to expose surface-residing bone cells as well as osteocytes to enhanced IFF. The capacity for these two mechanisms to generate spatially distinct flow profiles gives rise to the intriguing possibility that sensation of loading-induced IFF may occur differentially between osteocytes and surface-residing bone cells depending on the mechanism by which flow was generated, and that sensation of IFF generated via intramedullary pressure (ImP) may occur by a surface-residing bone cell population.

Though detailed descriptions of intramedullary pressurization-induced flow patterns at the endosteal surface have yet to be achieved, the specific magnitude and direction of endosteal IFF in response to intramedullary pressure (ImP) are expected to depend on a variety of factors such as the degree of heterogeneity in bone permeability as well as the mechanism by which ImP is generated. For example, uniform pressurization (such as that which would expected due to volumetric decreases in the marrow cavity [17]) is expected to give rise to largely radial flow due to the gross pressure gradient from the intramedullary compartment to the periosteal surface [18]. In this case, fluid passing from the marrow cavity into the LCS may pass through the cellular lining of the endosteal surface, giving rise to rise to shear stresses at endosteal intercellular junctions similar to those shown to stimulate vascular endothelial cells during transendothelial flow [20]. Flow tangential to the endosteal surface may also occur if local heterogeneities in bone permeability cause longitudinal or circumferential pressure gradients within the intramedullary compartment, or if the intramedullary compartment is pressurized in a non-uniform manner (for example, due to focal displacement of fluid arising from interactive effects between muscle activity and capillary filtration in bone tissue [13], [19]).

In a seminal study, Tatsumi and colleagues performed the first *in vivo* investigation of the effects of osteocyte depletion on bone mechanotransduction [21]. In particular, these authors generated transgenic mice (Dmp1-HBEGF) possessing the human gene for diphtheria toxin (DT) receptor fused to a 9.6 kb promoter sequence for dentin matrix protein 1 (Dmp1), allowing for inducible ablation of osteocytes upon administration of DT. Using this model, they were able to assess the effects of DT-induced osteocyte ablation on bone loss following hindlimb suspension (HLS) as well as recovery of bone mass following reloading. Surprisingly, while osteocyte ablation conferred resistance to trabecular bone loss induced by HLS, recovery of trabecular bone mass in transgenic mice following reloading occurred normally. Thus, while these studies confirmed a long-speculated mechanosensory function for osteocytes, they also strongly suggested the presence of one or more non-osteocytic mechanosensing cell populations within bone [21].

Given the capacity for intramedullary pressurization to differentially expose surface-residing bone cells to IFF, as well as emerging evidence suggesting the presence of one or more non-osteocytic mechanosensory bone cells, we speculated that adaptation to ImP-derived IFF may be mediated by a non-osteocytic bone cell population. To begin to explore this possibility, we investigated the effects of partial osteocyte ablation on structural adaptation to ImP-driven IFF. To induce pressure-driven IFF in the presence of minimal tissue strain, we used a recently developed microfluidic system for dynamically modulating ImP and IFF in alert mice [2]. In this system, peak cortical longitudinal strains during intramedullary pressure loading were found to be less than ∼10 µε [2] (implying that peak hoop strains were ∼20 µε or less as predicted by thin-walled pressure vessel theory). To deplete osteocytes, we used the Dmp1-HBEGF transgenic mouse model, which has been previously shown to give rise to partial ablation of osteocytes (as evidenced by 50% of lacunae being uninhabited, and 20% of inhabited lacunae containing osteocytes with apoptotic morphology) following a single administration of DT. Our findings indicate that structural adaptation in response to intramedullary pressurization-driven IFF is similar or significantly enhanced in mice with targeted osteocyte ablation, particularly in trabecular bone, despite up to ∼50% trabecular lacunae being uninhabited following ablation.

## Materials and Methods

### Ethics Statement

All animal procedures were in strict accordance with the NIH Guidelines for the Care and Use of Laboratory Animals and the guidelines of the Institutional Animal Care and Use Committee of the La Jolla Bioengineering Institute. The protocol for this study was approved by the LJBI IACUC (Animal Welfare Assurance No. A4343-01, Protocol No. PULS-101).

### Animals

C57BL/6Cr-Tg(Dmp1-HBEGF) mice were obtained from RIKEN BioResource Center (Tsukuba, Japan). Mice were maintained on a 12/12 hr light/dark cycle and given water and standard laboratory rodent chow *ad libitum*. For experiments, female wildtype (WT) and hemizygous transgenic (Tg) mice (16 weeks old) were administered 10 or 50 µg/kg DT (Sigma-Aldrich, St. Louis, MO) dissolved in PBS and administered i.p. These DT concentrations were selected based on the use of 2–50 µg/kg DT in Tg mice in previous studies [21]. Note that WT mice administered PBS or 50 µg/kg DT and Tg mice administered PBS were previously found by Tatsumi et al. to be phenotypically equivalent, exhibiting similar levels for percentage of empty lacunae, gene expression, trabecular bone loss following HLS, and recovery of trabecular bone mass following reloading [21].

### Cannulation and Microfluidic Enhancement of ImP

**Figure 1:**
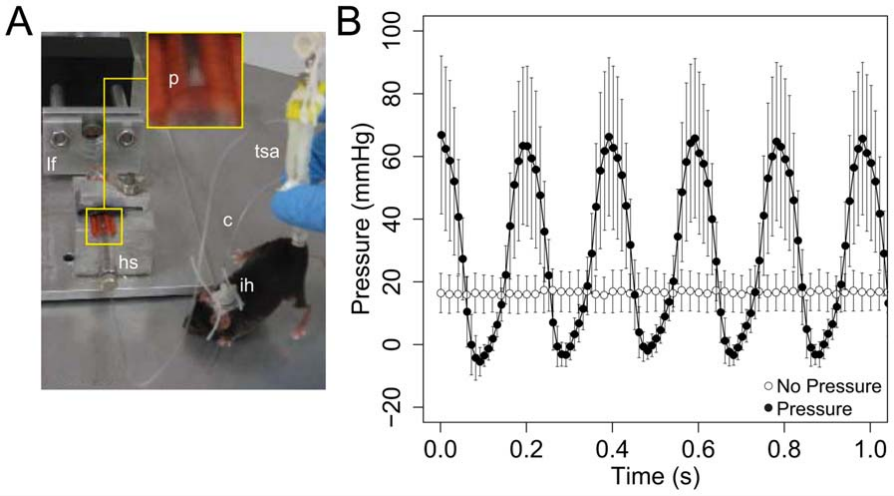
Experimental setup for pressure loading experiments and measurements of ImP. (A) Image of a hindlimb suspended mouse subjected to microfluidic pressure loading. The syringe pump consists of a Hamilton syringe (hs) mounted in a computer-controlled loading frame (lf) that actuates the syringe plunger (p). A saline-filled catheter (c) couples the pump to the cannulated mouse (hindlimb suspended via a tail suspension apparatus (tsa)). The catheter is protected from mouse chewing/pulling by an infusion harness (ih). (B) Composite average (± standard error) of intramedullary pressure measurements obtained from four animals in the absence (empty circles) and presence (filled circles) of microfluidic pressure loading. Pressure loading resulted in a 5.1 Hz waveform with a mean peak pressure of ∼70 mmHg.

A microfluidic system for modulating ImP was used to enhance IFF in alert mice as previously described [2]. Briefly, the distal femur was exposed in anesthetized mice, and a saline-filled catheter (0.5 mm outer diameter) was routed into the femoral intramedullary cavity through a drill hole ∼0.5 mm in diameter such that the tip was approximately 2 mm distal to the lesser trochanter (∼50 mm from the drill hole). The free end of the catheter was routed subcutaneously to the shoulder blades and passed through the skin through a small incision, and capped with a tubing plug. The external catheter end was protected from chewing/pulling by outfitting the mouse with an infusion harness [2], and passing the free catheter end through the access port. The use of right or left hindlimb for pressure loading was chosen randomly. The surgery was repeated on the contralateral limb; in this case the catheter was tied off tightly and remained subcutaneous for the duration of the experiment. Mice were administered analgesia and antibiotics as described [2]. Following surgery, all animals were observed to ambulate normally within 24 h of surgery. For pressure loading, the free end of the catheter was uncapped and coupled to an external computer-controlled microfluidic syringe pump (described in detail in [2]) (Fig. 1A). Mice were pressure-loaded using a 5.1 Hz oscillatory pressure profile with a pump stroke displacement of 0.5 µL, resulting in a peak pump flow rate of ∼5 µL/s. These parameters were similar to those used in previous studies [2], with the exception that the pump stroke displacement (and thus peak pump flow rate) were reduced by a factor of two. Measurement of ImP was performed using a telemetric pressure transducer placed at the mid-diaphysis as previously described [2]. Note that postmortem examination revealed no evidence of catheter slippage or leakage in any of the cannulated mice.

### Adaptation to HLS and Pressure Loading

Mice catheterized in both hindlimbs were used for all structural adaptation studies. Following a four-day recovery period, cannulated mice were subjected to pressure loading for 3 min/day for 14 days while under HLS or normal ambulation (Amb). HLS was carried out as described previously [2]. Mice were administered DT one day prior to the start of pressure loading, and a second administration one week later. Volumetric bone mineral density within a transverse section ∼1 mm thick at the lesser trochanter was quantified *in vivo* via pQCT (Stratec XCT Research SA+, Stratec, Birkenfeld, Germany) as described previously [2]. The lesser trochanter was selected due to previous studies demonstrating potent adaptive responses in trabecular and cortical trochanteric bone in response to both pressure loading and HLS [2]. Scanning was performed three days prior to cannulation (pre-loading) and at the end of the pressure loading period (post-loading). ΔBMD was defined as the difference between post- and pre-loading values; %ΔBMD was defined as the quotient of ΔBMD and pre-loading values. At the conclusion of experiments, femurs were obtained from euthanized animals and scanned using a μCT scanner (eXplore Locus, GE Healthcare, Waukesha, WI) within a transverse section ∼0.4 mm thick at the lesser trochanter [2]. Volumes of interest encompassing the entire cancellous compartment were constructed semi-automatically (MicroView, GE Healthcare, Waukesha, WI), and morphometry performed as previously described [2]. To compute cortical thickness, endocortical and periosteal surfaces were segmented semi-automatically every 0.1 mm in the z-direction using the freely available image segmentation software LiveWire [22]. Endocortical and periosteal areas were used to calculate effective periosteal and endocortical radii, and cortical thickness was computed as the average difference of effective radii [2].

### Genotyping

Genotyping was performed by PCR amplification of genomic tail DNA using the Extract-N-Amp Tissue PCR kit (Sigma-Aldrich, St. Louis, MO) according to the manufacturer's protocol [23]. To detect presence of the transgene, the following primers were used: 5′-CTGTATCCACGGACCAGCTGCTACC-3′ (forward) and 5′-CATGGGTCCCTCTTCTTCCCTAGC-3′ (reverse). For internal control, we used previously validated primers targeting PECAM-1 [23]: 5′-CAGCCACTGTGTGAGACACAAAGGCAAG-3′ (forward) and 5′-ACCACACACCCAGCAACCCTTTCAGAC-3′ (reverse).

### Histological Analysis

Empty lacunae were quantified in histological sections to assess the efficiency of osteocyte ablation [21]. Femurs were obtained from animals euthanized between 7–14 days following initial DT administration and immediately fixed in 10% neutral buffered formalin. H&E-stained longitudinal sections of paraffin-embedded decalcified bone (4 µm thick) were obtained from a commercial histopathology laboratory (Histo-Scientific Research Laboratories, Mount Jackson, VA). Histological analysis of trabecular and cortical lacunae was performed at the lesser trochanter, as this site was used for all assessments of bone mineral density and structural adaptation in HLS and pressure loading studies. Assessment was performed using a brightfield microscope equipped with a 10× objective and an analysis region of approximately 1 mm×0.2 mm in size for cortical bone and 1 mm×1 mm in size for trabecular bone. These dimensions were chosen since they were sufficiently large to view all cortical and trabecular bone within the lesser trochanter. Intracortical cavities (assessed as any cellularized cavity ∼50 µm or greater) were quantified in the same fields of view as that of cortical lacunae.

### *Ex Vivo* Measurement of Lacunar FRAP

*Ex vivo* measurements of fluorescence recovery after photobleaching (FRAP) within periosteal lacunae at the lesser trochanter were performed as previously described [2]. Briefly, cannulated mice were administered sodium fluorescein (300 mg/kg), euthanized, and their femurs quickly harvested. Bones were mounted in a custom holder and placed under a confocal laser scanning microscope in a dish filled with PBS warmed to 37°C. Individual lacunae on the periosteal surface were imaged and photobleached, and the recovery of fluorescence monitored every 32 s for 2 min. All experiments were performed within one hour of sacrifice. Fluorescence recovery rates were computed from the first three time points using the procedure described in [2]. For each lacuna, three image sequences were obtained using the pressure loading scheme: “no pressure loading”, “pressure loading”, “no pressure loading”. The “no pressure loading” recovery rate *k*_0_ was taken to be the average value obtained from the two “no pressure loading” image sequences [24]. An empirical relation derived from computational modeling of this process [25] was used to calculate peak canalicular flow velocity *v_c_*:
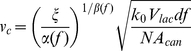
(1)where *ξ* = (*k*/*k*_0_)−1, *α*(*f*) and *β*(*f*) are functions of frequency whose values must be empirically determined at frequency *f*, *V_lac_* is the lacunar volume (calculated as 4π[*a* ^2^ *b*-(*a*-*w*)^2^(*b*-*w*)]/3, where *a* and *b* are the equatorial and polar radii, and *w* is the peak lacunar pericellular gap width), *d* is the canalicular length, *A_can_* is the canalicular cross-sectional area (calculated as π(*r*_o_^2^−*r*_i_^2^) where *r*_o_ and *r*_i_ are the canalicular and osteocyte process radii), and *N* is the number of contributing canaliculi. The following model parameters were assumed: *f* = 5 Hz, *α*(*f* = 5 Hz) = 1.5×10^−2^ [25], *β*(*f* = 5 Hz) = 1.7 [25], *N* = 11 [26], *r*_o_ = 130 nm [27], *r*_i_ = 52 nm [27], *a* = 4.0 µm [26], *b* = 8.6 µm [26], *w* = 0.5 µm [28], and *d* = 26 µm [26].

### Statistical Analysis

Statistical analysis was performed using multi-factorial ANOVA with genotype (WT or Tg), DT dose (10 or 50 µg/kg), and/or suspension (Amb or HLS) as factors (p<0.05 was considered statistically significant). For linear regression, we generated full first order models, and calculated the following values: Pearson correlation coefficient (r), multiple R-squared (R^2^), and adjusted R-squared (R_adjusted_^2^). The above statistical analyses were performed in the open source statistical environment R (http://www.R-project.org). To estimate statistical power, we used the fpower functionality in SAS (SAS Institute Inc., Cary, North Carolina), which computes power for one effect in a multifactorial ANOVA design. In this analysis, we assumed three factors of two levels each and an error level of α = 0.05. A pooled standard deviation was computed by pooling values across all groups. The effect size was assumed to be the lower of a) the greatest change observed between groups, or b) that which produced a power of 0.8. The sample size per treatment cell was assumed to be three (the minimum sample size used in this study) in order to provide the most conservative estimate of power. All values are reported as mean+/−standard error, with “WT” groups including wildtype animals administered 10 and 50 µg/kg DT, as no significant differences were detected between these two groups.

## Results

### Quantification of ImP and Lacunar-Canalicular Fluid Flow

**Figure 2:**
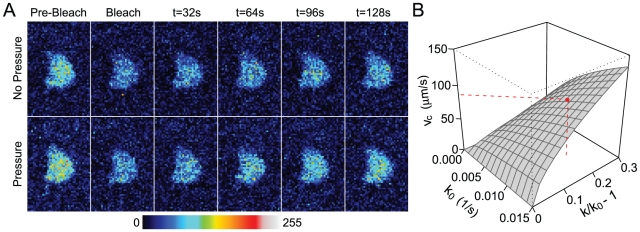
Quantification of canalicular convective velocity from *ex vivo* measurements of lacunar fluorescence recovery after photobleaching. (A) Single lacuna immediately prior to (Pre-Bleach) and following photobleaching (Bleach), and the subsequent recovery of fluorescence in the absence (top) and presence (bottom) of pressure loading. Faster recovery can be observed in the presence of pressure loading, indicating convective transport. Color bar on bottom indicates image intensity. (B) Plot of Eq. 1 demonstrating the relationship between convective velocity *v_c_* and recovery rates *k* and *k*_0_. The red dot corresponds to the canalicular fluid velocity (∼80 µm/s) calculated using the values of *k* and *k*_0_ obtained in this study.

Pressure loading resulted in a 5.1 Hz waveform that increased mean (no load: 16.1±6.4 mmHg, load: 27.0±9.6, n = 4 animals) and peak-to-peak (no load: 3.1±0.9 mmHg, load: 58.4±23.9 mmHg, n = 4 animals) ImP (Fig. 1B). To assess levels of flow within the LCS, we used an approach based on FRAP in which we quantified fluorescence recovery rates of sodium fluorescein within individual lacunae in the presence/absence (*k* and *k*_0_, respectively) of pressure loading (Fig. 2A) [2], [26]. We observed a *k*/*k*_0_ = 1.2±0.1 fold increase in recovery rate during pressure loading (*k*_0_: 0.011±0.002 s^−1^, *k*: 0.013±0.002 s^−1^, n = 9 lacunae from 4 animals), indicating enhanced solute transport due to convection [2], [24], [29]. Using Eq. 1, we found this corresponded to a peak canalicular flow velocity of ∼80 µm/s (Fig. 2B).

### Tg Mice Exhibit Compartment-Dependent Increases in Empty Lacunae Percentage

**Figure 3:**
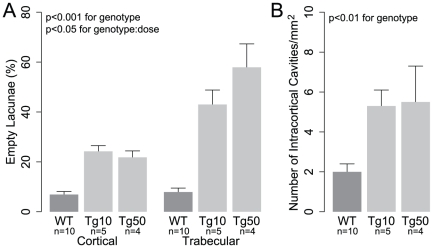
Transgenic mice exhibit significant increases in empty lacunae and intracortical cavities. (A) Quantification of empty lacunae in cortical and trabecular bone. (B) Quantification of intracortical cavities.

To assess efficiency of osteocyte ablation in Tg mice, we quantified empty lacunae in H&E-stained bone sections in both trabecular and cortical bone 7–14 days following initial DT administration (Fig. 3A). In cortical bone, Tg mice exhibited a significant increase in empty lacunae for both DT doses (WT: 6.9±1.2%, Tg10: 24.2±2.3%, Tg50: 21.8±2.6%, >2400 lacunae counted from n = 4–10 animals). Relative differences in these percentages (i.e., Tg10-WT and Tg50-WT) give the percentage of empty lacunae attributable to osteocyte ablation, and was calculated to be 15–17% for cortical bone. In trabecular bone, the presence of empty lacunae in Tg mice was substantially increased compared to that in cortical bone (WT: 7.9±1.6%, Tg10: 43.0±5.8%, Tg50: 57.9±9.4%, >400 lacunae counted from n = 3–10 animals). In this case, the percentage of empty lacunae attributable to osteocyte ablation was calculated to be 35–50%. Two-way ANOVA revealed a significant effect of genotype (p<0.001) and a significant genotype∶dose interaction (p = 0.02). We further assessed histological sections for intracortical cavities, which have been previously reported to be rapidly elevated following osteocyte ablation [21]. Consistent with these reports, we observed a significant effect of genotype (p = 0.01) in increasing intracortical cavities more than two-fold (WT: 2.0±0.4/mm^2^, Tg10: 5.3±0.8/mm^2^, Tg50: 5.5±1.8/mm^2^, >70 cavities counted from n = 4–10 animals) (Fig. 3B).

### Ambulating Tg Mice Exhibit Compromised Trabecular and Cortical Structure

To investigate the effects of osteocyte ablation on impairing bone homeostasis and compromising bone structure, we assessed structural indices in control limbs of ambulating WT and Tg mice. Consistent with previous studies demonstrating that targeted ablation of osteocytes results in rapid bone loss [21], we observed severely compromised trabecular and cortical structure and reduced bone mineral density in Tg mice relative to WT controls (Table 1). Tg mice exhibited significantly decreased trabecular volume fraction (Tg10 vs. WT: −8.9%; Tg50 vs. WT: −9.6%; p<0.01 for genotype), trabecular number (Tg10 vs. WT: −4.6%, Tg50 vs. WT: −16.6%, p<0.01 for genotype), and increased trabecular spacing (Tg10 vs. WT: 8.3%, Tg50 vs. WT: 26.8%, p<0.01 for genotype) compared to ambulatory WT controls. We did not detect a significant difference in trabecular thickness between WT and Tg mice (p = 0.79, power = 0.80 for detecting a difference of 0.005 mm). Tg mice also exhibited significantly decreased cortical thickness (Tg10 vs. WT: −7.2%, Tg50 vs. WT: −7.9%, p = 0.001 for genotype). With regard to bone mineral density, Tg mice administered 50 µg/kg DT (but not 10 µg/kg) displayed greater percentage losses in BMD than WT mice (Tg10 vs. WT: 1.0-fold, Tg50 vs. WT: 1.2-fold, p<0.01 for genotype). It is noteworthy we observed a reduction in BMD in ambulating WT mice (%ΔBMD in WT ambulating mice was ∼9%), potentially due to the invasiveness of the cannulation procedure and/or stress associated with daily pressure loading.

**Table 1:** Bone structure and bone mineral density in control limbs.

	BV/TVg,s		
	WT	Tg10	Tg50
**Amb**	22.8±0.9% (n = 6)	20.8±1.0% (n = 9)	20.6±2.6% (n = 3)
**HLS**	20.7±0.8% (n = 12)	18.1±0.8% (n = 11)	17.1±1.9% (n = 5)

	Tb.N^g^		
	WT	Tg10	Tg50
**Amb**	3.4±0.1 (n = 6)	3.2±0.2 (n = 9)	2.8±0.4 (n = 3)
**HLS**	3.7±0.2 (n = 12)	3.1±0.2 (n = 11)	2.7±0.2 (n = 5)

	Tb.Sp^g^		
	WT	Tg10	Tg50
**Amb**	0.23±0.01 mm (n = 6)	0.25±0.02 mm (n = 9)	0.30±0.04 mm (n = 3)
**HLS**	0.22±0.01 mm (n = 12)	0.27±0.02 mm (n = 11)	0.32±0.04 mm (n = 5)

	Tb.Th^s^		
	WT	Tg10	Tg50
**Amb**	0.068±0.001 mm (n = 6)	0.064±0.002 mm (n = 9)	0.073±0.005 mm (n = 3)
**HLS**	0.055±0.002 mm (n = 12)	0.057±0.001 mm (n = 11)	0.063±0.005 mm (n = 5)

	Ct.Thg,s		
	WT	Tg10	Tg50
**Amb**	0.191±0.004 mm (n = 6)	0.177±0.005 (n = 9)	0.176±0.005 mm (n = 3)
**HLS**	0.172±0.003 mm (n = 12)	0.169±0.003 mm (n = 11)	0.164±0.003 mm (n = 5)

	%ΔBMDg,s		
	WT	Tg10	Tg50
**Amb**	−8.8±0.8% (n = 6)	−8.7±1.1% (n = 9)	−10.2±1.6% (n = 3)
**HLS**	−11.9±0.8% (n = 12)	−16.4±0.6% (n = 11)	−14.4±0.7% (n = 5)

^g ^and ^s^ indicate statistically significant for genotype or suspension, respectively.

### HLS-Induced Bone Loss is Altered in Tg Mice in a Compartment-Dependent Manner

We next assessed the effects of osteocyte ablation in altering HLS-induced bone loss by comparing changes in structural indices in control limbs of hindlimb suspended mice relative to control limbs in ambulating animals (Table 1). In the trabecular compartment, Tg mice exhibited no significant reductions in HLS-induced losses in volume fraction (WT: −10.3%, Tg10: −14.9%, Tg50: −20.6%, p<0.01 for suspension, p = 0.71 for genotype∶suspension interaction, power = 0.80 for detecting a difference in volume fraction of 2.4%) or trabecular thickness (WT: −22.2%, Tg10: −12.4%, Tg50: −15.6%, p<0.001 for suspension, p = 0.17 for genotype∶suspension interaction, power = 0.80 for detecting a difference of 0.005). We did not observe a significant effect of suspension on trabecular number (WT Amb: 3.4±0.1, WT HLS: 3.7±0.2, p = 0.45 for suspension, power = 0.80 for detecting a difference of 0.3) or trabecular spacing (WT Amb: 0.23±0.01 mm, WT HLS: 0.22±0.01 mm, p = 0.79 for suspension, power = 0.12 for detecting a difference of 0.01 mm). In contrast to the lack of altered HLS-induced trabecular bone loss, Tg mice exhibited suppression of HLS-induced reductions in cortical thickness (WT: −11.3%, Tg10: −5.2%, Tg50: −7.2%, p<0.001 for suspension) in a manner that nearly reached statistical significance (p = 0.059 for genotype∶suspension interaction). In regard to bone mineral density, WT and Tg mice exhibited similar HLS/Amb fold changes in %ΔBMD (WT: 1.4-fold, Tg10: 1.9-fold, Tg50: 1.4-fold, p<0.001 for suspension), with no significant genotype∶suspension interaction observed (p = 0.054, power = 0.80 for detecting a difference of 2.2%).

### Pressure Loading-Induced Structural Adaptation is Enhanced in Tg Mice

**Figure 4:**
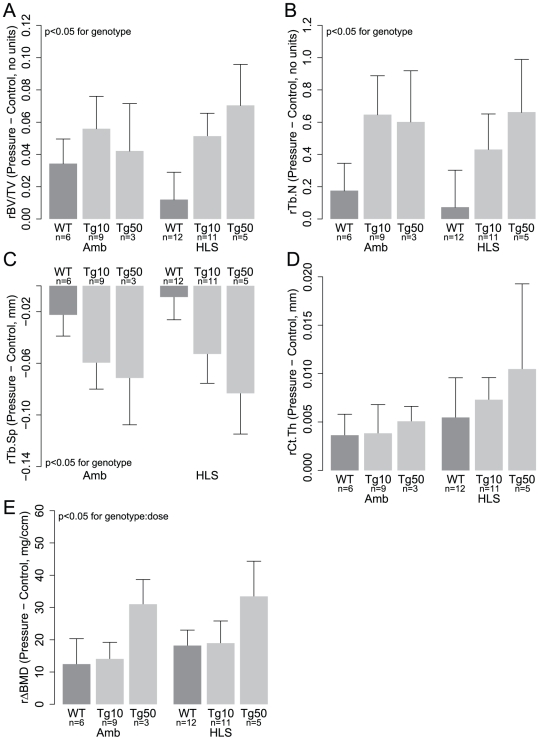
Pressure loading-induced adaptation is enhanced in transgenic mice. Results are shown for relative changes (defined as the difference between pressure-loaded limb and contralateral limb values) in (A) trabecular volume fraction, (B) trabecular number, (C) trabecular spacing, (D) cortical thickness, and (E) bone mineral density.

To assess effects of osteocyte ablation on structural adaptation to intramedullary pressurization-driven IFF, we quantified cortical and trabecular structure in pressure-loaded limbs in WT and Tg mice under HLS and normal ambulation. Interestingly, structural adaptation to ImP-driven IFF (as measured by relative gains between pressure-loaded and control limbs) was not abrogated and in most cases significantly enhanced in Tg mice. For example, compared to WT mice, hindlimb suspended Tg mice exhibited greater relative gains in trabecular volume fraction (WT: 1.2±1.7%, Tg10: 5.1±1.4%, Tg50: 7.0±2.5%) (Fig. 4A) trabecular number (WT: 0.1±0.2, Tg10: 0.4±0.2, Tg50: 0.7±0.3) (Fig. 4B), and trabecular spacing (WT: −0.01±0.01 mm, Tg10: −0.05±0.02 mm, Tg50: −0.08±0.03 mm) (Fig. 4C). Hindlimb suspended Tg mice also exhibited increased relative gains in cortical thickness (WT: 5.5±4.1 µm, Tg10: 7.3±2.3 µm, Tg50: 10.5±8.8 µm) (Fig. 4D) and ΔBMD (WT: 18.2±4.8 mg/ccm, Tg10: 18.9±6.9 mg/ccm, Tg50: 33.4±10.9 mg/ccm) (Fig. 4E). Similar trends were observed in ambulatory mice for trabecular volume fraction (WT: 3.4±1.5%, Tg10: 5.6±2.0%, Tg50: 4.2±2.9%) (Fig. 4A), trabecular number (WT: 0.2±0.2, Tg10: 0.6±0.2, Tg50: 0.6±0.3) (Fig. 4B), trabecular spacing (WT: −0.02±0.02 mm, Tg10: −0.06±0.02 mm, Tg50: −0.07±0.04 mm) (Fig. 4C), cortical thickness (WT: 3.6±2.2 µm, Tg10: 3.8±3.0 µm, Tg50: 5.1±1.6 µm) (Fig. 4D), and ΔBMD (WT: 12.4±7.9 mg/ccm, Tg10: 14.1±5.1 mg/ccm, Tg50: 33.1±7.6 mg/ccm) (Fig. 4E). A significant effect of genotype in increasing relative gains was found for all indices of trabecular structure (p<0.05 for all cases) but not cortical thickness (p = 0.54, power = 0.11 for detecting a difference of 1.5 µm). The enhancement of ΔBMD was significant in both a genotype- and DT dose-dependent manner (genotype∶dose interaction: p<0.05). For relative gains in trabecular thickness, animals subjected to ambulation (WT: 6.5±2.5 µm, Tg10: 3.7±3.3 µm, Tg50: −0.6±3.7 µm) and HLS (WT: 1.8±2.5 µm, Tg10: 7.1±2.1 µm, Tg50: 8.6±4.0 µm) exhibited opposite trends in regard to effects of genotype. No significant effect of genotype was observed (p = 0.53, power = 0.8 for detecting a difference of 4.6 µm).

### Control Limb Structure Explains Intra- and Inter-group Variability in Adaptation to Pressure Loading

**Figure 5:**
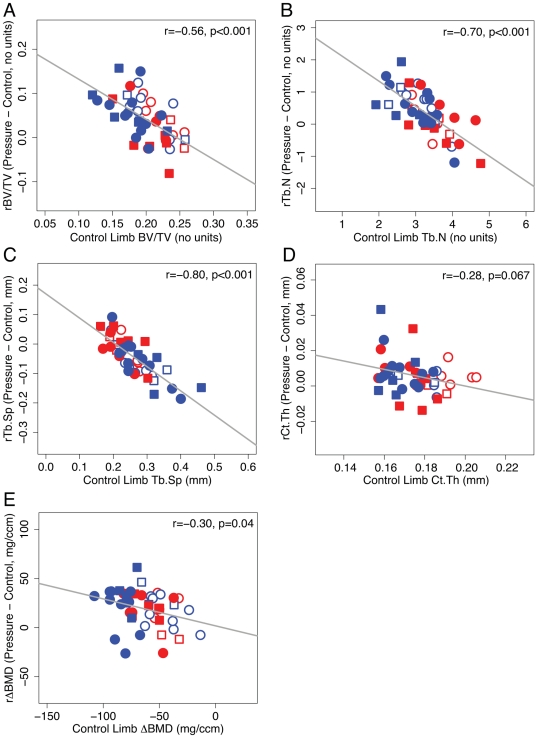
Bone structure in control limb predicts inter- and intra-group variability in adaptation to pressure loading. Bone structure in control limb is plotted in the x-coordinate, and is indicative of baseline cellular activity in the absence of pressure loading. Relative adaptation is plotted in the y-coordinate, and was found to be negatively correlated with control limb structure independently of genotype and DT dose. Results are shown for (A) trabecular volume fraction, (B) trabecular number, (C) trabecular spacing, (D) cortical thickness, and (E) bone mineral density. Each point represents a single animal (red: WT; blue: Tg; circle: 10 µg/kg DT; square: 50 µg/kg DT; fill: HLS; no fill: Amb). Pearson correlation coefficients and corresponding p-values are shown in the top right of each plot.

We next performed linear regression analyses to assess whether adaptive responses in pressure-loaded limbs were correlated with control limb structure. Our rationale for performing these analyses was based on three observations. First, changes in control limb structure are manifested by alterations in osteoblastic and/or osteoclastic activity incurred independently of pressure loading (e.g., as a result of osteocyte ablation and or HLS), and thus are an indicator of pressure loading-independent changes in osteoblastic and/or osteoclastic activity. Second, the potential to undergo pressure loading-induced adaptation is determined in large part on the local cellular environment at the time of loading, such as the number of mechanically-sensitive cells, and their baseline level of activity. Finally, the biochemical alterations driving changes in osteoblastic and/or osteoclastic activity that are incurred as a result of pressure loading-independent stimuli (e.g., HLS and/or osteocyte ablation) have a high likelihood to occur in both limbs of each animal. Thus, we speculated that correlations may exist between adaptive responses in pressure-loaded limbs and control limb structure, with positive correlations indicating potentiation of pressure loading-induced adaptation by baseline increases in osteoblastic activity and/or decreases in osteoclastic activity, and negative correlations indicating the opposite. To test this, we performed linear regression analysis for all structural indices using control limb values as the predictor variable and relative gains in pressure-loaded limbs as the dependent variable. Interestingly, for trabecular bone volume fraction, number, and spacing, we found that gains in pressure-loaded limbs demonstrated marked and highly significant negative correlations with control limb values (BV/TV: r = −0.56, p<0.001; Tb.N: r = −0.70, p<0.001; Tb.Sp: r = −0.80, p<0.001) independently of specification of genotype, suspension condition, or DT dose (Fig. 5). Bone mineral density and cortical thickness also exhibited negative though weaker correlations that were statistically significant (ΔBMD: r = −0.30, p = 0.04) or nearly significant (Ct.Th: r = −0.28, p = 0.067). For the highly correlated indices obtained from the trabecular compartment (BV/TV, Tb.N, and Tb.Sp), control limb values alone were found to explain 35∼64% of the observed variability in adaptation in pressure-loaded limbs (BV/TV: R^2^ = 0.35, R_adjusted_^2^ = 0.33; Tb.N: R^2^ = 0.49, R_adjusted_^2^ = 0.48; Tb.Sp: R^2^ = 0.64, R_adjusted_^2^ = 0.63). It is noteworthy that adding genotype, suspension condition, and DT dose as predictor variables in the model did not increase R_adjusted_^2^, which corrects for the addition of variables that are uncorrelated with the dependent variable being modeled (BV/TV: R_adjusted_^2^ = 0.31; Tb.N: R_adjusted_^2^ = 0.48; Tb.Sp: R_adjusted_^2^ = 0.62).

## Discussion

Mechanical loading generates skeletal IFF via multiple mechanisms that result in spatially distinct flow profiles. While poroelastic interactions within the LCS result in flow primarily within the lacunar-canalicular network in which osteocytes reside, dynamic pressurization of the intramedullary cavity results in significant flow into and out of the marrow cavity, potentially exposing surface-residing cells to enhanced flow in addition to osteocytes. Given the emerging evidence that bone possesses multiple mechanosensory cellular populations, we speculated that adaptation to ImP-derived IFF may be sensed by surface residing cells independently and potentially parallel to sensation of poroelasticity-derived flow via osteocytes. To begin to explore this possibility, we assessed the effects of partial osteocyte ablation in altering cortical and trabecular structural adaptation to dynamic intramedullary pressure loading. By directly pressurizing the intramedullary cavity, we were able to simulate the flow patterns that would be expected to occur in the presence of dynamic increases in ImP (e.g., due to loading-induced volumetric changes in the intramedullary compartment [17], or interactive effects between muscle activity and capillary filtration [13], [19]) while avoiding confounding effects associated with matrix deformation.

During intramedullary pressure loading, we used a loading profile that generated fluid displacements and peak pressures similar in magnitude to those expected to occur during impact loading but greater than those expected to occur during ambulation. For example, the peak rate of change in marrow cavity volume during physiological loading can be estimated by finding the product of peak longitudinal strain rate (0.1 s^−1^ and 0.03 s^−1^ during impact and ambulation respectively [30]) and marrow cavity volume (10 µL [31]). We found that the peak pump flow rate used in our studies (∼5 µL/s) was similar to the peak rate of change in marrow cavity volume expected to occur during impact loading (1 µL/s) but greater than that expected to occur during ambulation (0.3 µL/s). It is notable that this flow rate was half the 10 µL/s previously shown by Kwon et al. to induce peak-to-peak pressures double that observed in this study (∼60 mmHg vs. ∼120 mmHg), suggesting a linear change in peak ImP with pump flow rate over the range spanned by the two studies.

Telemetric pressure measurements revealed peak ImP was on the order of ∼70 mmHg. Though *in vivo* measurements of ImP in the mouse femur during impact loading have yet to be attained, previous studies suggest that the peak pressures generated in this study were similar to those generated during impact but greater than those generated during ambulation. For example, this pressure is below the ∼300 mmHg found to occur during simulated impact in sheep tibia *in vitro* [32] but similar to pressures generated *in vivo* during step compression in turkey ulna (600 µε, ∼65 mmHg) [17] and low-strain electrical muscle stimulation in rat femur (100 µε, ∼45 mmHg) [13]. The pressures generated in this study are much greater than those expected to occur during ambulation. For example, Stevens et al. observed an approximately 2 mmHg decrease in ImP in the mouse femur upon going from ambulation to hindlimb suspension [33], more than 30-fold less than the mean peak ImP observed in this study. It is noteworthy that pump displacements were calibrated to generate peak pressures substantially below ∼100 mmHg in order to mitigate the potential to stimulate cells via fluid pressure. In particular, to the authors' knowledge, no studies to date have demonstrated the capacity for bone cells to sense pressures less than 97.5 mmHg [34]. In addition, for the short duration of loading used here (3 min/day), *in vitro* studies suggest peak pressures several-fold larger than those generated in this study are required to result in bone cell stimulation [2], [35], [36].

To quantify levels of IFF induced by intramedullary pressurization, we monitored FRAP recovery rates in periosteal lacunae [2], [26]. We previously determined an empirical relationship between recovery rates *k* and *k*_0_ and peak canalicular convective flow velocity using a computational model based on the convection-diffusion equation and the geometry of the pericellular fluid space within the LCS [25], [37]. Using this relationship and values of *k* and *k*_0_ obtained in this study, we estimated peak canalicular convective velocities of ∼80 µm/s. Recently, peak canalicular velocities of ∼60 µm/s [29] were estimated in mouse tibiae under compressive strains (∼400 µε) similar to those found to occur during jump loading (∼600 µε) [38]. This suggests that in our studies, flow within the LCS was being generated at physiological levels, and the inability for osteocyte ablation to abrogate structural adaptation to pressure loading was not attributable to insufficient generation of lacunar-canalicular IFF.

To confirm successful osteocyte ablation, we quantified empty lacunae in cortical and trabecular bone. We found osteocyte ablation resulted in a significant number of empty lacunae, with the percentage attributable to osteocyte ablation greater in trabecular bone (up to ∼50%) compared to cortical bone (up to ∼17%). This trend was similar to previous studies in which Tatsumi et al. observed greater numbers of empty lacunae in trabecular versus cortical bone (60% in trabecular bone, 50% in cortical bone) [21], suggesting that Dmp1-driven DTR expression may be more ubiquitous in the trabecular compartment. Overall, ablation efficiency in our study appeared to be lower compared to that in the studies of Tatsumi et al, particularly in cortical bone. This may be in part attributable to lower expression of the Dmp1 promoter in the mature (16 week-old mice in our studies) versus immature skeleton (empty lacunae were quantified in 10 week-old mice in the studies of Tatsumi et al., though unloading studies were performed in 20 week-old mice), particularly given the role of Dmp1 in promoting mineralization [39], [40] and hydroxyapatite formation (e.g., see [41]). However, it is important to note that the increases in empty lacunae observed in this study are likely to be reflective of a higher percentage of actual osteocytes ablated. In particular, Tatsumi et al. previously observed Tg mice possessed a significant number of inhabited lacunae containing apoptotic osteocytes (∼40%) and thus concluded that in mice in which 50% of lacunae were uninhabited, 50+(50*.40) = ∼70% osteocytes were ablated in total.

In the absence of pressure loading, we found that ambulating Tg mice exhibited rapid and severe deterioration of trochanteric cortical and trabecular structure. The rapid (i.e., within 14 days of DT administration) cortical bone loss was consistent with the intracortical resorption and cortical thinning in Tg mice previously observed to occur within eight days following DT administration [21]. However, we also observed rapid deterioration in trochanteric trabecular structure, in contrast to Tatsumi et al. who found normal levels of trabecular volume fraction in the distal femur two weeks following DT administration but substantial losses after 40 days [21]. Importantly, these data suggest that in our studies, osteocyte ablation itself induced a much greater degree of resorptive activity in trabecular bone compared to the studies of Tatsumi et al. In addition, these data indicate that short-term resistance to osteocyte ablation-induced bone loss is not a specific trait of trabecular bone, but instead may be sensitive to site-specific factors such as local cellular makeup and biochemical environment that may differ between anatomical sites (such as the distal femur and lesser trochanter). It is noteworthy that though we observed modest losses in BMD in ambulating WT mice, these losses were likely due to effects associated with the cannulation procedure rather than a response to DT itself. In particular, we have previously observed some losses in BMD in ambulating WT mice in unpressurized limbs in the absence of DT administration (data not shown). In addition, previous studies indicate that DT is insufficient to alter osteoblastic and osteoclastic activity in the absence of the transgene [21].

When Tg mice were subjected to hindlimb unloading, we observed differential alteration of HLS-induced cortical and trabecular structural deterioration. In particular, Tg mice exhibited reduced losses in cortical thickness but similar losses in trabecular structure. While the reduced cortical thinning is consistent with findings of Tatsumi et al. demonstrating that osteocyte ablation inhibits HLS-induced decreases in bone mass, the similar or greater extent of trabecular bone loss suggests that the capacity for osteocyte ablation to inhibit this process may be highly sensitive to a variety of factors such as the local cellular makeup and activity levels prior to HLS. For example, as discussed earlier, in our studies there was a much greater degree of trabecular bone loss in response to osteocyte ablation itself compared to the studies of Tatsumi et al. Thus, we speculate that the inability for osteocyte ablation to inhibit HLS-induced trabecular bone loss in our studies may be attributable to higher levels of resorptive activity as a result of osteocyte ablation, which may have masked any reduction in osteocyte-derived osteoclastogenic signals during HLS that occur following osteocyte ablation. In regard to bone mineral density, WT and Tg mice exhibited similar HLS-induced changes in %ΔBMD, however it is important to note that that the resolution of the pQCT scanner (0.1 mm nominal resolution) is much greater than the changes in cortical thickness quantified via μCT (∼10 µm). In this case, any differences in HLS-induced losses in cortical thickness would likely not be reflected in BMD measurements. Given that the scanner resolution is similar to the thickness of cortical bone (∼0.2 mm) but substantially greater than the thickness of individual trabeculae (∼50 µm), changes in BMD likely reflect alterations in trabecular volume fraction, intracortical porosity, or changes in cortical and/or trabecular tissue mineral density.

In pressure-loaded limbs, we found that structural adaptation to ImP-driven IFF was similar or enhanced in cortical bone and in most cases significantly enhanced in trabecular bone in Tg mice. Interestingly, enhancement was particularly evident in the trabecular compartment, despite up to ∼50% of trabecular lacunae being uninhabited following ablation. Though the capacity for adaptation to ImP-driven IFF to proceed unimpaired following a 15–50% decrease in viable osteocytes is consistent with the hypothesis that sensation of ImP-driven IFF is mediated by a non-osteocytic bone cell population, it is important to consider potential roles of the reduced population of viable osteocytes in the adaptation process. For example, the lack of impairment in adaptation in Tg mice may have evolved out of hyper-stimulation of the reduced non-ablated osteocyte population, such as could arise from increases in fluid flow due to increased lacunar-canalicular permeability. However, if the enhanced adaptation in Tg mice was attributable to hyper-stimulation of the reduced non-ablated osteocyte population, one would expect a highly non-linear relationship between enhancement of adaptation and ablation percentage due to the competing effects of reduced numbers of mechanosensing cells and potentiation of their mechanosensitivity by ablation (e.g., enhancement at low ablation percentages, but abrogation at higher percentages). In this case, our data suggests this phenomenon was not occurring, as we observed a wide range of ablation percentages (as low as 15% and high as 50% empty lacunae) to be ineffective in abrogating pressure loading-induced adaptation.

Several other potential mechanisms may explain the inability for partial osteocyte ablation to abrogate the adaptive response to intramedullary pressure loading that do not preclude the involvement of osteocytes in a mechanosensory role. For example, it has been put forth that osteocytes may mediate this process in an antagonistic role by functioning as a cellular thermostat, halting bone formation initiated by mechanical loading once a sufficiently dense osteocytic network has been formed. Such a role for osteocytes would be consistent with recent studies demonstrating that deletion of osteoblastic and osteocytic gap junctions enhances load-induced bone formation [42]. Though we did not assess for osteocytes in newly formed bone in pressure-loaded mice, previous studies indicate that DT-induced ablation of osteocytes is a transient and reversible process, as evidenced by the long-term restoration of bone mass and osteocyte numbers to pre-ablation levels [21]. Another possibility is that the number of osteocytes required for sensation of ImP-induced IFF is normally excessive, and that reduction of the population by 50% is insufficient to prevent ImP-induced bone adaptation. Further studies investigating pressure loading-induced adaptation in the presence of more complete osteocyte depletion are required in order to explore these questions further. However, such studies would require the development of an alternative mouse model that allows for more efficient osteocyte ablation, as to the authors' knowledge, no such model currently exists.

**Figure 6:**
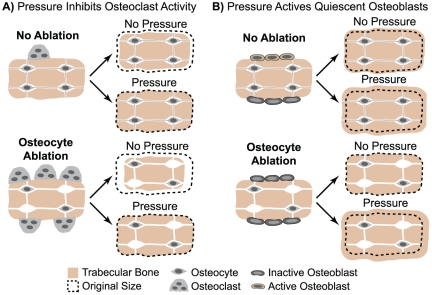
Schematic demonstrating two potential mechanisms by which osteocyte ablation may give rise to loss of trabecular bone mass in unpressurized limbs while enhancing pressure loading-induced adaptation. In the first case (A), osteocyte ablation gives rise to an increase in the number of active osteoclasts, resulting in heightened bone loss in unpressurized limbs. In limbs subjected to pressure loading, the resorptive activity of these active osteoclasts is halted, preserving bone mass. In the second case (B), osteocyte ablation shifts the osteoblastic population to a more quiescent state, resulting in decreased bone mass in unpressurized limbs. However, in pressure-loaded limbs, an enhanced anabolic response occurs due to the newly available pool of quiescent cells activated following exposure to pressure loading-induced IFF.

In linear regression analysis, we found marked and highly significant negative correlations between control limb and pressure-loaded limb structure, with no functional improvement in the model when genotype, DT dose, and suspension condition were added as predictors. The existence of an inverse correlation between unpressurized limb bone structure and responsiveness to pressure loading suggests that within each animal, changes in the cellular environment incurred independently of pressure loading (i.e., as a result of osteocyte ablation and/or HLS) occurred in both limbs, and that these cellular changes had the simultaneous effect of decreasing bone mass in control limbs while increasing the potential to undergo pressure loading-induced adaptation. Interestingly, previous studies suggest that flow-induced inhibition of osteoclastic activity may occur via direct sensation of IFF by osteoclasts or their precursors [6], or indirectly via flow-induced down-regulation of osteoclastogenic factors in marrow stromal cells [7]. In addition, direct stimulation of osteoblasts by fluid flow has also been widely demonstrated [4]. Based on these studies, we propose two potential mechanisms by which changes in the cellular environment following osteocyte ablation could simultaneously decrease control limb bone structure while enhancing the potential to undergo adaptation to pressure loading (Fig. 6). In the first case (Fig. 6A), osteocyte ablation gives rise to an increase in the number of active osteoclasts, resulting in heightened bone loss in unpressurized limbs. In limbs subjected to pressure loading, the resorptive activity of these active osteoclasts is halted, preserving bone mass. In the second case (Fig. 6B), osteocyte ablation shifts the osteoblastic population to a more quiescent state, resulting in decreased bone mass in unpressurized limbs. However, in pressure-loaded limbs, a heightened anabolic response occurs due to the newly available pool of quiescent cells that can be activated by ImP-induced IFF. In both cases, the net result is bone loss in the control limb but enhanced adaptation to pressure loading in Tg mice.

The unexpected capacity for partial osteocyte ablation to enhance adaptation to pressure loading motivates future studies exploring the mechanisms underlying this phenomenon, potentially through the use of molecular biology and/or alternative genetic approaches. For example, the use of an alternative promoter sequence for driving osteocytic DTR expression (such as the Sost promoter [43]) may enable more ubiquitous osteocyte ablation, allowing for the assessment of pressure loading-induced adaptation in the presence of near-complete osteocyte depletion. In addition, the use of immunohistochemistry for the detection of early signaling pathway activation [44] or *in situ* hybridization for the detection of early response gene expression [45] would allow for the identification of specific cell populations stimulated soon after exposure to pressure loading. Mouse models of inducible osteoblast ablation [46] may be used to explore the effects of osteoblastic depletion on adaptation to pressure loading. Finally, knockout mouse models with bone-specific deletion of gap junctional proteins such as connexin 43 [42] may be useful in elucidating the role of impaired osteocyte network signaling in pressure loading-induced adaptation.

Several limitations should be considered when interpreting the findings from this study. First, our study primarily focused on structural outcomes since they are the most clinically relevant measures. However, they do not provide definitive insight as to whether the response was anabolic or anti-resorptive (or both) in nature. While we previously established that pressure loading increases endosteal mineral apposition and bone formation rate [2], future studies which assess osteoclast number and activity in parallel with osteoblast number, activity, and dynamic histomorphometric measures will be valuable in providing insight into the anabolic versus anti-resorptive nature of the enhanced adaptation observed in Tg mice. Second, our experimental design was primarily constructed to detect effects of genotype rather than DT dose. For example, in most cases we were unable to detect significant DT dose∶genotype interactions in pressure loading studies, despite some evidence of dose-dependent trends. This may be attributable to the low number of Tg mice administered 50 µg/kg DT relative to those administered 10 µg/kg, which was dictated by initial studies in which we found the former group to exhibit greater enhancement of pressure loading-induced adaptation (and thus less animals to detect differences relative to WT controls). However, it is noteworthy that our primary objective was to assess effects of genotype in pressure loading-induced adaptation, and in this regard our study was sufficiently powered to detect significant effects of genotype and/or suspension in most cases. A final limitation is that the cannulation procedure used in this study is associated with inflammation and coagulation, giving rise to concerns that effects associated with these processes may be driving bone adaptation rather than IFF. However, it is important to note that all animals were cannulated in both hindlimbs, minimizing the potential for adaptation in pressure-loaded limbs to be attributable to inflammatory processes associated with the cannulation procedure. In addition, we have previously observed similar adaptive responses to pressure loading when using heparinized and unheparinized saline within the catheters (data not shown), suggesting that adaptation to pressure loading was not dependent on the formation of intra-catheter clots.

In conclusion, our studies indicate that bone structural adaptation to intramedullary pressurization-driven IFF is similar or significantly enhanced in mice with targeted osteocyte ablation, particularly in trabecular bone, despite up to 50% of trabecular lacunae being uninhabited following ablation. These exploratory data are consistent with the potential existence of non-osteocytic mechanosensory bone cells that sense ImP-driven IFF independently and potentially parallel to osteocytic sensation of poroelasticity-derived IFF within the LCS. An emerging body of evidence indicates that in addition to osteocytes, bone may possess non-osteocytic mechanosensory cells that may be activated during mechanical loading. Identification of these bone cell populations and the mechanisms by which they are stimulated in mechanically loaded bone are exciting possibilities for future investigations that have yet to be fully explored.
